# The complete mitochondrial genome data of *Tonna sulcosa* (Born, 1778) (Gastropoda: Tonnidae) from China

**DOI:** 10.1016/j.dib.2026.113295

**Published:** 2026-09-29

**Authors:** Dewei Cheng, Teng Huang, Xin Liu, Qunjian Yin, Ersha Dang

**Affiliations:** a Guangxi Laboratory of Oceanography (GXLO), Beihai, 536015, China; b Key Laboratory of Tropical Marine Ecosystem and Bioresource, Fourth Institute of Oceanography, Ministry of Natural Resources, Beihai, 536015, China; c Guangxi Key Laboratory of Beibu Gulf Marine Resources, Environment and Sustainable Development, Fourth Institute of Oceanography, Ministry of Natural Resources, Beihai, 536015, China

**Keywords:** *Tonna sulcosa*, Mitochondrial genome, Phylogenetic analysis, Marine organism

## Abstract

Tonnoidea are ecologically significant marine predatory gastropods, while mitogenomic data are still scarce for many members of this superfamily. We assembled the complete circular mitochondrial genome of *Tonna sulcosa* (Born, 1778) using hybrid Illumina-Nanopore sequencing. A total of 35,574,310 paired-end Illumina short reads and 1478,546 Nanopore long reads were generated, yielding average sequencing depths of 853.64 × (Illumina) and 9391.60 × (Nanopore), respectively. The 17,348 bp mitogenome contains 37 typical metazoan mitochondrial genes: 13 protein-coding genes, 22 tRNAs and two rRNAs. Phylogenetic analysis based on 13 concatenated mitochondrial PCGs from 38 sequences of 37 gastropod species showed that Tonnoidea forms a well-supported monophyletic group. Two *T. sulcosa* mitogenomes (this study and OP696777.1) are nested within Tonnidae and cluster with *T. dolium* and *T. galea*, supporting the monophyly of genus *Tonna*. This mitogenome supplies useful data for future evolutionary studies of Tonnoidea gastropods.

Specifications Table

**Table:** 

Subject	Biology
Specific subject area	Mitogenomics and Phylogeny
Type of data	Raw sequencing reads (FASTQ), assembled mitochondrial genome (FASTA), annotated genome file (GFF/GenBank format)
Data collection	Genomic DNA source: An individual specimen (voucher: TS-1) Genomic DNA isolation: TIANamp Marine Animals DNA Kit (Tiangen, Beijing, China) Library construction: Illumina TruSeq Nano DNA Sample Prep Kit and Oxford Nanopore library preparation kit Sequencing: Illumina NovaSeq 6000 (150 bp paired‑end) and Oxford Nanopore PromethION (long‑read) Quality check: NanoDrop 2000, Illumina Experiment Manager Assembly and annotation: miniasm v0.3‑r179, NextPolish v1.3.1, Bowtie2 v2.3.5.1, Unicycler v0.4.8, MITOS2, MitoZ, Geneious Prime v2021.0.3 Mitogenome map construction: ProkSee. (https://proksee.ca/) Phylogenetic tree analysis: PhyloSuite v1.2.3, MAFFT v7.471, trimAl v1.2, ModelFinder, IQ‑TREE v2.2.0 tRNA analysis: MITOS2 / MitoZ (integrated annotation)
Data source location	Institution:Fourth Institute of Oceanography, Ministry of Natural Resources City/Town/Region: Beihai, Guangxi Country: China Latitude and longitude (and GPS coordinates, if possible) for collected samples/data: Latitude: 20°3′8.02′’ N, Longitude: 109°5′13.78′’ E)
Data accessibility	Repository name: NCBI Sequence Read Archive (SRA) Data identification number: SRR39289980 and SRR39289981 Direct URL to data: https://www.ncbi.nlm.nih.gov/sra/SRR39289980 https://www.ncbi.nlm.nih.gov/sra/SRR39289981 Repository name: NCBI BioProject Data identification number: PRJNA1480968 Direct URL to data: https://www.ncbi.nlm.nih.gov/bioproject/PRJNA1480968 Repository name: NCBI BioSample Data identification number: SAMN61047942 Direct URL to data: https://www.ncbi.nlm.nih.gov/biosample/SAMN61047942 Repository name: NCBI GenBank Data identification number: PZ517662 Direct URL to data: https://www.ncbi.nlm.nih.gov/nuccore/PZ517662
Related research article	Not applicable

## 1 Value of the Data

• This dataset provides an independently assembled complete mitochondrial genome of *T. sulcosa*, addressing the lack of detailed structural and non‑coding‑region annotations for this species despite a previously released mitogenome entry (GenBank: OP696777.1) in public repositories.
• The data can be reused by researchers conducting phylogenetic and evolutionary studies within Tonnoidea and the broader Caenogastropoda, and supports intraspecific mitogenomic comparison against the earlier published OP696777.1 sequence.
• The hybrid sequencing approach (Illumina and Nanopore) provides high-quality assembly data that can serve as a reference for future mitogenomic studies of other tonnid species.

## 2 Background

Tonnoidea constitutes a moderately speciose superfamily within Caenogastropoda, encompassing approximately 361 valid marine species distributed across 21 families worldwide [1]. Its members exhibit a cosmopolitan marine distribution ranging from intertidal habitats to bathyal depths, with the vast majority inhabiting tropical and subtropical warm marine waters [2]. Despite their ecological significance and extensive morphological variation, the systematic classification and internal phylogenetic relationships within Tonnoidea remain incompletely resolved and require further molecular validation.

Mitochondrial genomes are widely recognized as reliable and efficient molecular markers for exploring the phylogeny and evolutionary patterns of marine invertebrates [3,4]. In recent years, mitogenomic studies focusing on Tonnoidea have substantially increased to resolve its ambiguous internal taxonomy and evolutionary relationships. Previous investigations have covered multiple representative species across different tonnoidean families, conducting comprehensive analyses of mitogenomic characteristics, sequence variation, and adaptive evolution [[5], [6], [7]]. These available mitogenomic resources have laid a preliminary foundation for understanding the evolutionary framework of Tonnoidea; however, molecular data focusing specifically on the family Tonnidae remain relatively scarce.

The mitochondrial genomic information of *Tonna sulcosa* (Born, 1778) (Fig. 1), a common large gastropod widely distributed in the tropical Indo-Pacific Ocean, was previously reported in a Tonnoidea‑focused mitogenomic study, with a mitogenome deposited under GenBank accession OP696777.1 [6]. Although OP696777.1 was generated for superfamily-level phylogenetic reconstruction, it lacks detailed annotation and characterization of non-coding regions, including the putative control region. Analyses concerning gene arrangement, nucleotide skew, codon usage and intraspecific variation for this species are still absent. The absence of publicly accessible genomic data greatly hinders subsequent comparative mitogenomic analyses, taxonomic verification, and in-depth evolutionary exploration of Tonnidae. Overall, current mitogenomic resources for Tonnidae remain insufficient compared with those for other families within Tonnoidea.

**Fig. 1:**
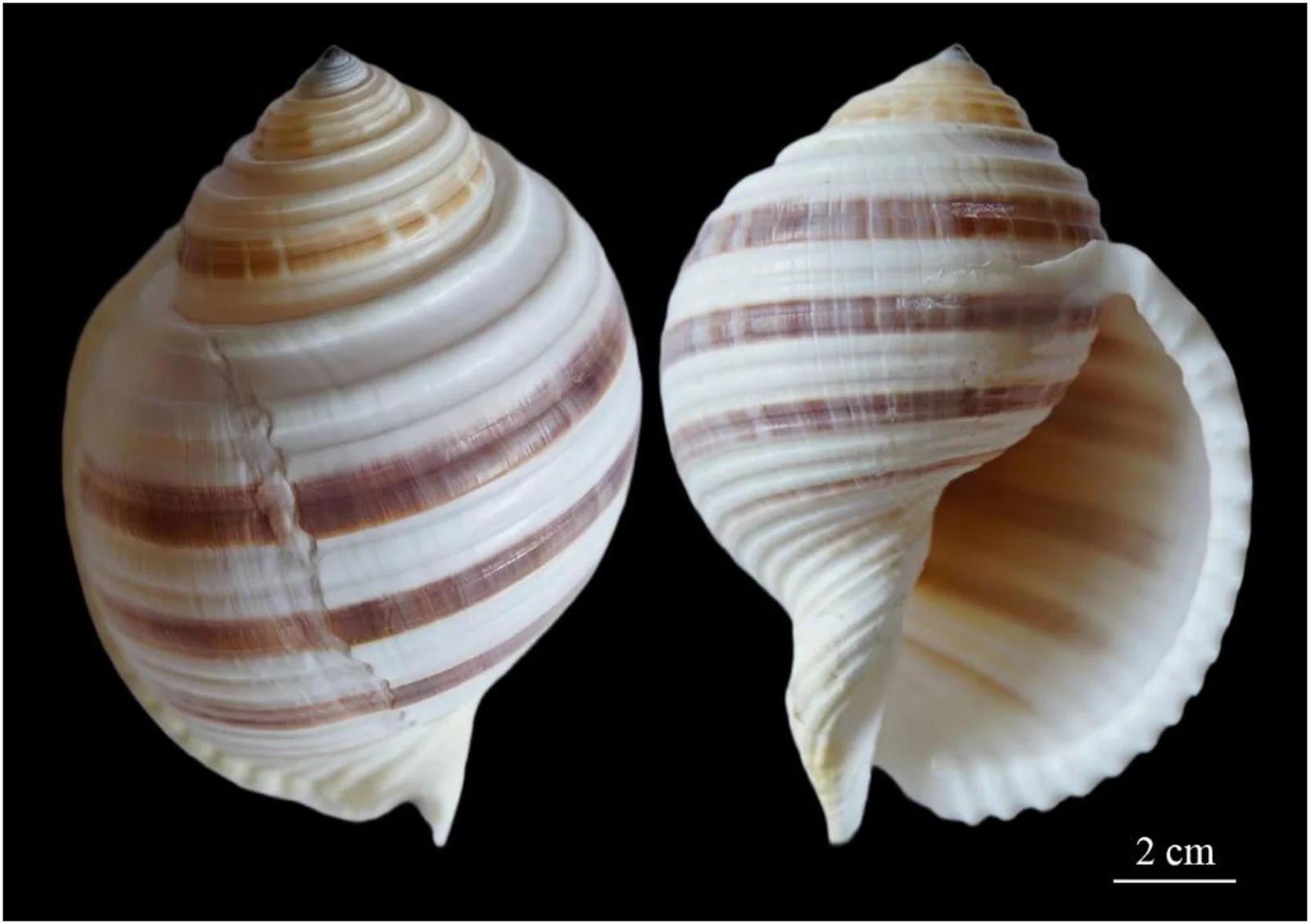
Apertural and dorsal morphological views of the voucher specimen TS‑1 of *T. sulcosa*, collected in June 2023 from the Beibu Gulf, China. This individual was used for DNA extraction and mitochondrial genome sequencing. Scale bar = 2 cm. Photograph by Dewei Cheng.

To fill this critical data gap, the present study performed full-length mitochondrial genome sequencing, assembly, and annotation of *T. sulcosa* using a hybrid Illumina and Nanopore sequencing strategy. Compared with OP696777.1, our hybrid-based assembly yields a more complete mitogenome and explicitly characterizes the large putative A+T-rich control region. The complete mitochondrial genome and raw sequencing data obtained in this study were formally reported here. This newly established genomic dataset provides valuable fundamental molecular resources for future phylogenetic reconstruction, taxonomic revision, and comparative mitogenomic analysis of Tonnoidea and Caenogastropoda.

## 3 Data Description

### 3.1 Characteristics of the *T. sulcosa* mitochondrial genome

The complete mitochondrial genome of *T. sulcosa* was successfully sequenced and assembled, yielding a closed circular mitochondrial DNA molecule with a total length of 17,348 bp (NCBI GenBank accession: PZ517662.1). Hybrid sequencing produced 35,574,310 paired-end Illumina raw short reads and 1478,546 Nanopore raw long reads. The average sequencing depth was 853.64 × for Illumina short reads and 9391.60 × for Nanopore long reads; Depth‑distribution profiles for both sequencing platforms are shown in Supplementary Fig. S1. Consistent with the typical gene composition of metazoan mitochondrial genomes [8], the assembled *T. sulcosa* mitogenome encodes 37 functional genes, including 13 protein-coding genes (PCGs), 22 transfer RNA (tRNA) genes, and two ribosomal RNA (rRNA) genes (Fig. 2). Furthermore, the gene arrangement pattern of *T. sulcosa* is highly conserved and identical to that of previously reported species within the superfamily Tonnoidea [[5], [6], [7]].

**Fig. 2:**
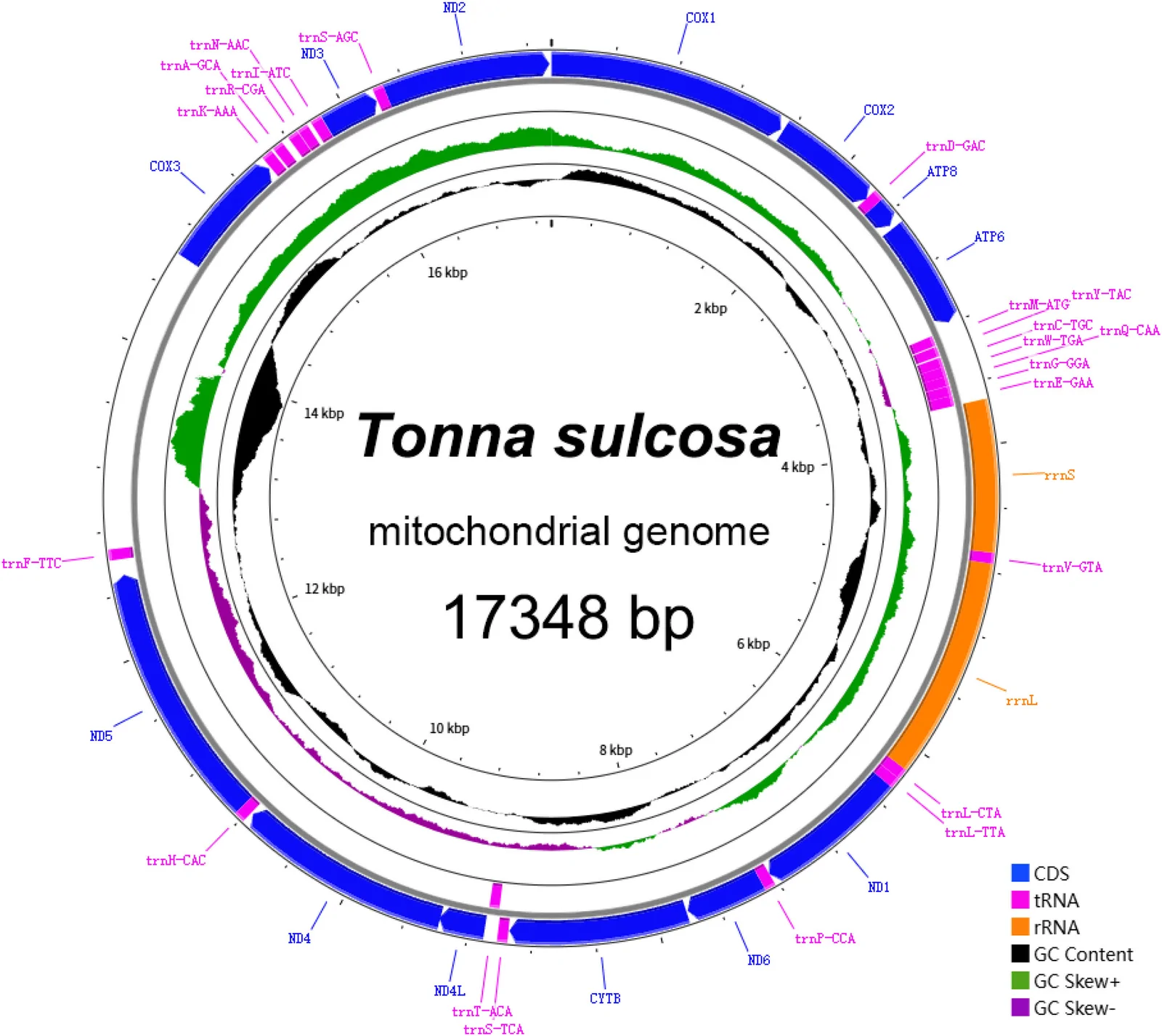
Mitochondrial genome map of *T. sulcosa* (GenBank accession number: PZ517662). Genes are color-coded according to their functional classification. Blue represents 13 PCGs, pink represents 22 tRNAs, yellow represents 2 rRNAs. The black color in the inner ring indicates GC content, while the green and purple represent GC skew.

Nucleotide composition analysis revealed that the *T. sulcosa* mitochondrial genome exhibits a pronounced AT bias, with an overall A+T content of 72.2% (32.7% A and 39.5% T), while the G and C contents are 15.0% and 12.8%, respectively. Strand compositional asymmetry was further confirmed by skew analysis, with the entire mitochondrial genome presenting a negative AT skew (−0.094) and a positive GC skew (0.079). Initiation and termination codon usage of the 13 PCGs was systematically summarized in Table 1. Consistent with most gastropod mitochondrial genomes, nearly all PCGs utilize the ATG start codon, whereas the ND4 gene is an exception with a GTG initiation codon. Twelve PCGs terminate with conventional canonical stop codons (TAA or TAG). Gene strands were annotated as forward (+) heavy strand and reverse (−) light strand. Among the 22 annotated tRNA genes of *T. sulcosa*, eight are encoded on the reverse (−) light strand, and the remaining 14 tRNAs are located on the forward (+) heavy strand, with gene lengths ranging from 65 bp to 71 bp. In addition, the two rRNA genes (12S rRNA: 962 bp; 16S rRNA: 1398 bp) are spatially separated by the trnV-GTA gene in the mitochondrial genome. A large non‑coding intergenic gap between trnF‑TTC and COX3 (positions 12,694–14,612, 1919 bp) represents the putative mitochondrial control region (A+T‑rich region), which is the main non‑coding segment in this mitogenome and is consistent with the mitogenomic features of related gastropod species. This region has been listed in Table 1. Relative synonymous codon usage (RSCU) analysis of the 13 mitochondrial protein‑coding genes revealed a strong A/U‑ending codon bias in *T. sulcosa*. A total of 3721 sense codons were counted, of which 29 codons showed RSCU values > 1. The codon UUA (Leu) exhibited the highest RSCU value (3.8021) and also represented the most‑frequently used codon (365 occurrences). All codons with RSCU > 1 ended in A or U, whereas C‑ or G‑ending codons had RSCU values below 1. This pattern is consistent with the high A/T compositional bias observed in this mitogenome. RSCU statistics and codon counts are summarized in supplementary Table S1 and visualized in supplementary Fig. S2.

**Table 1:** Characteristics of the mitochondrial genome of *T. sulcosa*. Direction indicates forward (+) and reverse (−) strands.

Start (bp)	End (bp)	Length (bp)	Strand	Initial codons	Terminal codons	Gene
1	1536	1536	+	ATG	TAA	COX1
1554	2240	687	+	ATG	TAA	COX2
2239	2306	68	+			trnD-GAC
2308	2466	159	+	ATG	TAA	ATP8
2492	3187	696	+	ATG	TAA	ATP6
3226	3293	68	-			trnM-ATG
3302	3367	66	-			trnY-TAC
3379	3444	66	-			trnC-TGC
3445	3511	67	-			trnW-TGA
3511	3575	65	-			trnQ-CAA
3578	3644	67	-			trnG-GGA
3645	3715	71	-			trnE-GAA
3716	4677	962	+			rrnS
4678	4745	68	+			trnV-GTA
4746	6143	1398	+			rrnL
6144	6212	69	+			trnL-CTA
6214	6282	69	+			trnL-TTA
6283	7224	942	+	ATG	TAG	ND1
7225	7293	69	+			trnP-CCA
7295	7795	501	+	ATG	TAA	ND6
7805	8944	1140	+	ATG	TAA	CYTB
8950	9014	65	+			trnS-TCA
9025	9092	68	-			trnT-ACA
9101	9397	297	+	ATG	TAG	ND4L
9391	10,767	1377	+	GTG	TAG	ND4
10,781	10,849	69	+			trnH-CAC
10,850	12,523	1674	+	ATG	TAG	ND5
12,625	12,693	69	+			trnF-TTC
12,694	14,612	1919	+			Putative control region (A+T-rich non-coding region)
14,613	15,392	780	+	ATG	TAG	COX3
15,405	15,475	71	+			trnK-AAA
15,491	15,558	68	+			trnA-GCA
15,607	15,675	69	+			trnR-CGA
15,680	15,747	68	+			trnN-AAC
15,776	15,846	71	+			trnI-ATC
15,850	16,203	354	+	ATG	TAG	ND3
16,210	16,277	68	+			trnS-AGC
16,278	17,336	1059	+	ATG	TAA	ND2

### 3.2 Phylogenetic analysis

Phylogenetic analysis of *T. sulcosa* was conducted based on the concatenated nucleotide sequences of 13 mitochondrial protein-coding genes (PCGs) from 38 sequences representing 37 gastropod species, including the previously published *T. sulcosa* mitogenome (GenBank: OP696777.1) for intrasspecific comparison. Both Maximum-likelihood (ML) and Bayesian Inference (BI) phylogenetic reconstructions were performed in this study. The reconstructed phylogenetic topology from ML and BI analyses are largely congruent, and all Tonnoidea species form a well-supported monophyletic clade (Fig 3, Fig 4). Within Tonnoidea, two *T. sulcosa* sequences (OP696777.1 and PZ517662.1) cluster tightly together and are nested within Tonnidae, grouping with congeners *T. dolium* and *T. galea.* This result further validates the monophyly of the genus *Tonna* at the mitochondrial genomic level.

**Fig. 3:**
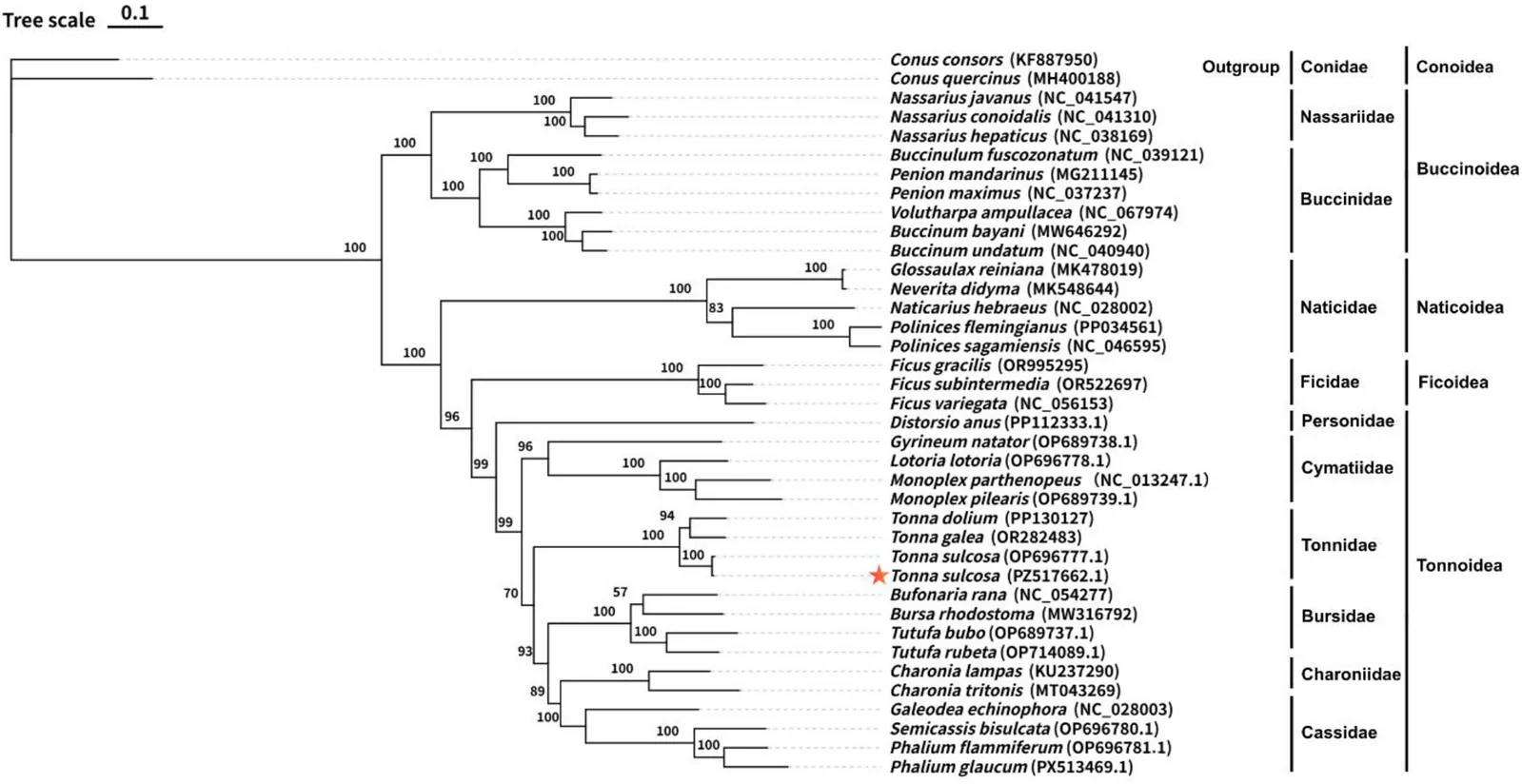
Maximum-likelihood (ML) phylogenetic tree was reconstructed based on the concatenated 13 protein-coding genes of 38 sequences representing 37 species. Accession numbers were indicated after the species name. Numbers at the nodes indicated bootstrap support value from 1000 replicates. The red star indicates the *T. sulcosa* sequence obtained in this study.

**Fig. 4:**
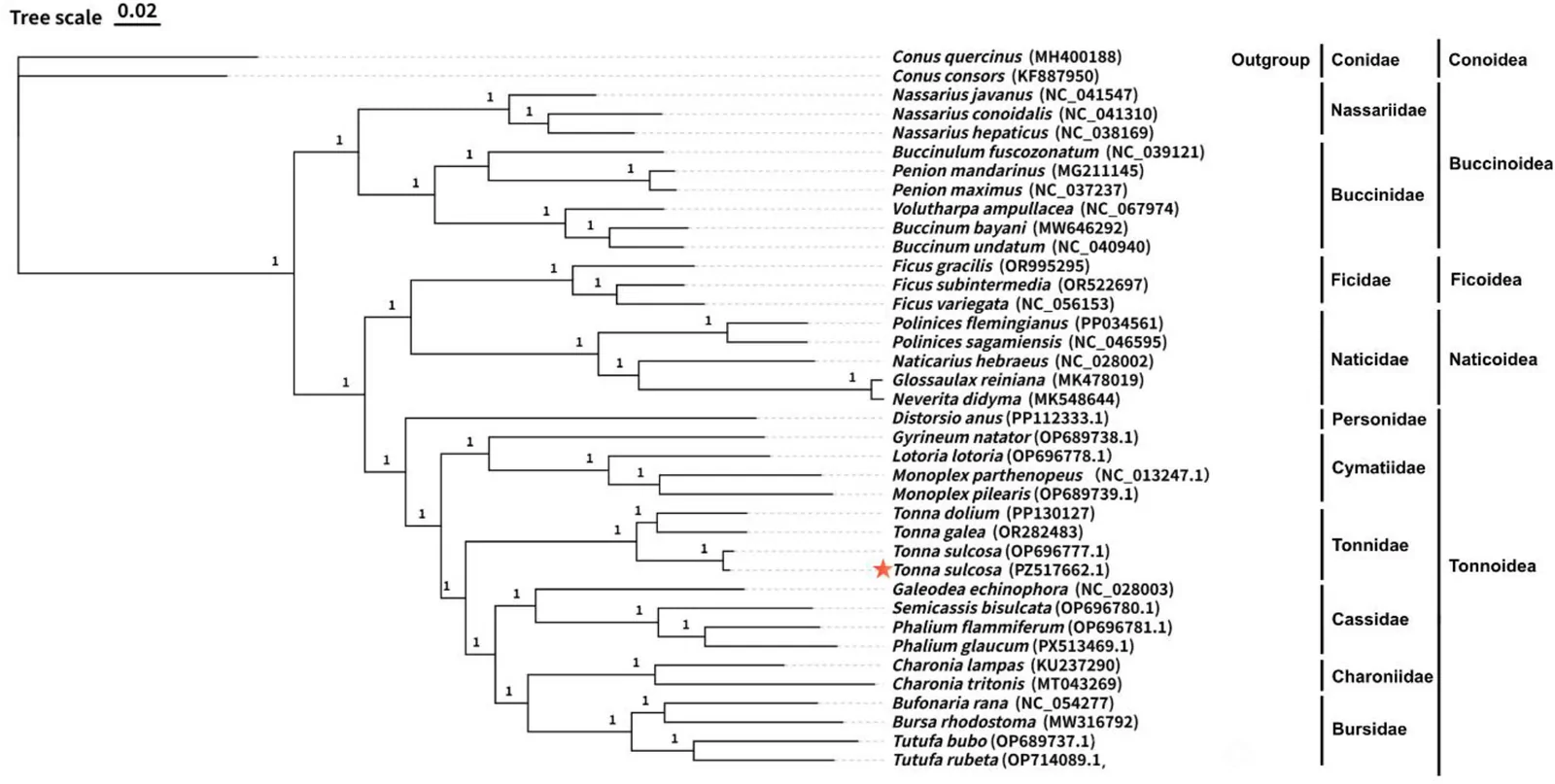
Bayesian inference (BI) phylogenetic tree reconstructed based on the concatenated 13 protein-coding genes from two *T. sulcosa* mitogenomes and 36 other species. GenBank accession numbers are indicated after the species names. Numbers at the nodes indicate Bayesian posterior probabilities, the scale bar represents 0.02 substitutions per site. The red star indicates the *T. sulcosa* sequence obtained in this study.

Regarding the internal phylogenetic architecture of Tonnoidea (Fig 3, Fig 4), Cymatiidae and Personidae form the basal lineage of Tonnoidea, followed by Tonnidae. Tonnidae is sister to the clade composed of Bursidae, Charoniidae and Cassidae, rather than forming a sister-group relationship with Ficidae. Ficidae falls outside the Tonnoidea clade in both phylogenetic trees, consistent with recent mitogenomic investigations that place Ficidae within Ficoidea [9]. Overall, the mitogenome-based phylogenetic results unambiguously resolve the evolutionary position of *T. sulcosa* within Tonnoidea and provide solid molecular evidence for the taxonomic delimitation and evolutionary research of tonnoidean gastropods.

## 4 Experimental Design, Materials and Methods

### 4.1 Sample collection

A mature individual of *T. sulcosa* was collected via commercial trawling in the Beibu Gulf, China (109°5′13.78″ E, 20°3′8.02″ N). Adductor muscle tissue was dissected from the specimen and immediately fixed in 95% ethanol for subsequent molecular experiments. The voucher specimen (catalog ID: TS-1) is permanently deposited in the Laboratory of the Fourth Institute of Oceanography, Ministry of Natural Resources, China. Sample inquiries can be addressed to DW Cheng (dwcheng2022@163.com).

### 4.2 DNA extraction, sequencing and mitochondrial genome assembly

Genomic DNA was isolated from ethanol-fixed muscle samples with the TIANamp Marine Animals DNA Kit (Tiangen, Beijing, China), strictly operating in accordance with the manufacturer’s standard protocols. The concentration and A260/A280 purity ratio of extracted DNA were quantified via a NanoDrop 2000 spectrophotometer (Thermo Fisher Scientific, Massachusetts, USA).

A hybrid long-read and short-read sequencing strategy was adopted for mitogenome data acquisition. Genomic libraries were constructed and sequenced on two platforms: Oxford Nanopore PromethION (Oxford Nanopore Technologies, Oxford, UK) for long-read data generation and Illumina NovaSeq 6000 (Illumina, San Diego, USA) for high-accuracy paired-end short reads.

High-quality clean reads filtered from both platforms were used for *de novo* assembly of the complete mitochondrial genome. A preliminary draft assembly was generated using miniasm v0.3 r179 [10] and further error-corrected with NextPolish v1.3.1. Clean Illumina short reads were mapped to the polished draft assembly via Bowtie2 v2.3.5.1 [11]. Finally, hybrid assembly integrating Illumina short reads and Nanopore long reads was completed using Unicycler v0.4.8 [12]. Sequencing depth across the circular mitogenome was calculated separately for Illumina and Nanopore reads by mapping clean reads against the final assembled mitogenome.

### 4.3 Mitogenome annotation and sequence analysis

The assembled circular mitogenome was subjected to gene annotation and sequence-feature characterization. Initial gene prediction was performed with MITOS2 [13] and MitoZ [14]. The results were integrated and manually curated in Geneious Prime v2021.0.3, where protein‑coding gene boundaries were verified via open‑reading‑frame analysis against closely related tonnid mitogenome references. Finally, the circular nature and integrity of the final *T. sulcosa* mitochondrial genome assembly were confirmed and visualized using Proksee [15], and nucleotide skewness was assessed using the equations: AT skew = (A‑T)/(A+T); GC skew = (G‑C)/(G+C) [16]. Relative synonymous codon usage (RSCU) was calculated for each of the 13 protein‑coding genes under the NCBI invertebrate mitochondrial genetic code. Stop codons (TAA and TAG) were excluded from the calculation. RSCU was computed following the formula reported by Sharp et al. [17]. Calculation and data validation were implemented by custom scripts in R (v4.5.1) [18]. The visualization was produced using ggplot2 [19] with the viridis colour palette [20], and plot panels were assembled with the patchwork package [21].

### 4.4 Phylogenetic analysis

Maximum-likelihood (ML) and Bayesian Inference (BI) phylogenetic reconstruction was performed following the analytical framework described by Zheng et al. [6], and all sequence processing procedures were implemented in PhyloSuite v1.2.3 [22]. The dataset comprised concatenated sequences of 13 mitochondrial PCGs from 38 sequences representing 37 gastropod taxa, with the newly generated *T. sulcosa* mitogenome as the focal sample. Two *Conus* species (*C. consors* and *C. quercinus*) were selected as outgroups, following the rooting strategy widely applied in previous Tonnoidea phylogenetic studies [6].

Within the PhyloSuite workflow, individual PCG sequences were independently aligned using MAFFT v7.471 [23], and ambiguous conserved sites were trimmed using trimAl v1.2 [24]. ModelFinder [25] was used to determine the optimal partition scheme and nucleotide substitution models based on the Bayesian Information Criterion (BIC). The final ML phylogenetic tree was constructed in IQ-TREE v2.2.0 [26] with an edge-linked partition model, and node support was evaluated via 1000 bootstrap replicates. Bayesian phylogenetic analysis was executed in MrBayes v3.2.7 [27], running four independent Markov chain Monte Carlo (MCMC) chains for 2 × 10⁶ generations. Trees were sampled every 100 generations, with the initial 25 % of samples discarded as burn-in. The remaining trees were used to calculate posterior probabilities for each node.

## Limitations

Only a single individual specimen (TS‑1) of *T. sulcosa* was sequenced in this work, and all analyses are restricted to mitochondrial DNA data. Comprehensive genome‑wide population‑level comparison, detailed structural variant detection and selection‑pressure analyses are beyond the scope of this Data‑in‑Brief data‑description article; such dedicated comparative genomic analyses will be addressed in future primary‑research publications. More geographical population samples are required to fully uncover intraspecific mitogenomic polymorphism of *T. sulcosa*.

## Ethics Statement

The authors have read and followed the ethical requirements for publication in Data in Brief. The authors confirm the current work does not involve human subjects, animal experiments, or any data collected from social media platforms.

## CRediT Author Statement

**Dewei Cheng**: Conceptualization, data curation, Formal analysis, Investigation, Writing-original draft; **Teng Huang**: Data curation, Investigation, Methodology; **Xin Liu**: Investigation, Validation; **Qunjian Yin:** Resources, Investigation; **Ersha Dang**: Conceptualization, Funding acquisition, Project administration, Resources, Supervision, Validation, Writing-review & editing. All authors have approved the final version of manuscript and all authors agree to be accountable for all aspects of the work.

## Appendix Supplementary materials

Image, image 1 dummy alt text

Image, image 2 dummy alt text

Image, application 3

Image, application 4
