# Supplementary material for: The complete mitochondrial genome data of Tonna sulcosa (Born, 1778) (Gastropoda: Tonnidae) from China
Source: Data Brief. 2026 Sep 29;69:113295. doi: 10.1016/j.dib.2026.113295 (PMC13673022; doi:10.1016/j.dib.2026.113295)
Supplement: Supplementary file 1 [file mmc3.docx]

**Supplementary Fig. S1.** Sequencing‑depth distribution of the *T. sulcosa* mitochondrial genome. A. Illumina sequencing, B. Nanopore sequencing. X-axis represents genomic position (bp); Y-axis represents sequencing depth (×).

**Supplementary Fig. S2.** Analysis of relative synonymous codon (RSCU) in the *T. sulcosa* mtDNA. The X-axis and Y-axis represent the types of amino acids and their RSCU values, respectively. Each amino acid, which can be encoded by multiple codons, is presented in different colored histograms.
