# Supplementary material for: The complete mitochondrial genome data of Tonna sulcosa (Born, 1778) (Gastropoda: Tonnidae) from China
Source: Data Brief. 2026 Sep 29;69:113295. doi: 10.1016/j.dib.2026.113295 (PMC13673022; doi:10.1016/j.dib.2026.113295)
Supplement: Supplementary file 2 [file mmc4.docx]

**Supplementary Table S1.** Relative synonymous codon usage and codon number of *T. sulcosa* mitochondrial PCGs

| **Codon** | **No.** | **RSCU** | **Codon** | **No.** | **RSCU** | **Codon** | **No.** | **RSCU** |
| --- | --- | --- | --- | --- | --- | --- | --- | --- |
| GCA(A) | 93 | 1.55 | CUA(L) | 62 | 0.6458 | AGG(S) | 11 | 0.2372 |
| GCC(A) | 33 | 0.55 | CUC(L) | 5 | 0.0521 | AGU(S) | 47 | 1.0135 |
| GCG(A) | 7 | 0.1167 | CUG(L) | 10 | 0.1042 | UCA(S) | 85 | 1.8329 |
| GCU(A) | 107 | 1.7833 | CUU(L) | 80 | 0.8333 | UCC(S) | 17 | 0.3666 |
| UGC(C) | 6 | 0.3077 | UUA(L) | 365 | 3.8021 | UCG(S) | 13 | 0.2803 |
| UGU(C) | 33 | 1.6923 | UUG(L) | 54 | 0.5625 | UCU(S) | 100 | 2.1563 |
| GAC(D) | 18 | 0.4675 | AUA(M) | 173 | 1.6168 | ACA(T) | 63 | 1.44 |
| GAU(D) | 59 | 1.5325 | AUG(M) | 41 | 0.3832 | ACC(T) | 20 | 0.4571 |
| GAA(E) | 67 | 1.5581 | AAC(N) | 19 | 0.3016 | ACG(T) | 16 | 0.3657 |
| GAG(E) | 19 | 0.4419 | AAU(N) | 107 | 1.6984 | ACU(T) | 76 | 1.7371 |
| UUC(F) | 42 | 0.2642 | CCA(P) | 57 | 1.5405 | GUA(V) | 96 | 1.6134 |
| UUU(F) | 276 | 1.7358 | CCC(P) | 12 | 0.3243 | GUC(V) | 17 | 0.2857 |
| GGA(G) | 111 | 1.7903 | CCG(P) | 10 | 0.2703 | GUG(V) | 16 | 0.2689 |
| GGC(G) | 15 | 0.2419 | CCU(P) | 69 | 1.8649 | GUU(V) | 109 | 1.8319 |
| GGG(G) | 32 | 0.5161 | CAA(Q) | 69 | 1.7692 | UGA(W) | 102 | 1.8378 |
| GGU(G) | 90 | 1.4516 | CAG(Q) | 9 | 0.2308 | UGG(W) | 9 | 0.1622 |
| CAC(H) | 19 | 0.475 | CGA(R) | 33 | 2.2 | UAC(Y) | 17 | 0.2394 |
| CAU(H) | 61 | 1.525 | CGC(R) | 2 | 0.1333 | UAU(Y) | 125 | 1.7606 |
| AUC(I) | 20 | 0.1316 | CGG(R) | 3 | 0.2 | / | / | / |
| AUU(I) | 284 | 1.8684 | CGU(R) | 22 | 1.4667 | / | / | / |
| AAA(K) | 70 | 1.5556 | AGA(S) | 82 | 1.7682 | / | / | / |
| AAG(K) | 20 | 0.4444 | AGC(S) | 16 | 0.345 | / | / | / |

**Note:** RSCU was calculated from 3,721 sense codons. TAA and TAG were excluded. Display values are rounded to four decimal places; calculations used unrounded codon counts. '/' denotes an empty layout slot.
